# Immune-modulatory effect of human milk in reducing the risk of Kawasaki disease: A nationwide study in Korea

**DOI:** 10.3389/fped.2022.1001272

**Published:** 2022-09-08

**Authors:** Jae Yoon Na, Yongil Cho, Juncheol Lee, Seung Yang, Yong Joo Kim

**Affiliations:** ^1^Department of Pediatrics, Hanyang University Hospital, Seoul, South Korea; ^2^Department of Emergency Medicine, Hanyang University College of Medicine, Seoul, South Korea; ^3^Department of Emergency Medicine, Hanyang University Hospital, Seoul, South Korea; ^4^Department of Pediatrics, Hanyang University College of Medicine, Seoul, South Korea

**Keywords:** Kawasaki disease, mucocutaneous lymph node syndrome, breastfeeding, national data study, immunomodulatory effect

## Abstract

**Background:**

Kawasaki disease (KD) is the most common acquired heart disease among children in developed countries, but the etiology is still unclear. There are several hypotheses regarding the outbreak of KD, including infection, genetics, and immunity. Since breastfeeding plays an essential role in the immune system's composition, investigating breastfeeding's effects on the occurrence of KD would be an excellent way to identify the etiology of KD.

**Aim:**

To determine whether the incidence of KD decreases with breastfeeding.

**Methods:**

This nationwide cohort study analyzed data from the National Health Insurance Service (NHIS) in South Korea and included 1,910,438 infants who underwent their first National Children's Health Examination (NCHE) between 2008 and 2014. Feeding types were collected using a questionnaire in NCHE. The NHIS data and NCHE data were merged and analyzed. First, we investigated the effect of breastfeeding on the development of KD at 1 year of age. Then, we surveyed the age at which no significant effect appeared by expanding the observation range yearly.

**Results:**

The most prevalent feeding type in the study population was exclusive breastfeeding (41.5%). At 10–12 months of follow-up age, 3,854 (0.2%) infants were diagnosed with KD. Compared to the exclusive formula feeding group, the adjusted odds ratio (aOR) for KD was 0.84 [95% confidence interval (CI), 0.78–0.90] and 0.86 (95% CI, 0.79–0.94) in the exclusive and partial breastfeeding groups, respectively. At 22–24 months of age, aOR for KD was 0.94 (95% CI, 0.90–0.98) in the exclusive breastfeeding group and 0.98 (95% CI, 0.92–1.03) in the partial breastfeeding group. There was no difference in the risk between the groups at 34–36 months.

**Conclusions:**

Using a large amount of national data on children aged <2 years, we proved that breastfeeding has a protective effect on the development of KD.

## Introduction

Kawasaki disease (KD) has gained increasing attention due to its clinical similarity with the multisystem inflammatory syndrome in children related to coronavirus disease 2019 (COVID-19) infection. KD is the most common acquired heart disease among children in developed countries and is a systemic vasculitis that invades small- and medium-sized arteries, especially the coronary arteries (1, 2). Coronary aneurysms can occur in approximately 20–25% of patients with KD who have not received adequate treatment and result in fatal complications, such as myocarditis, arrhythmia, and even sudden death (3). Despite numerous studies, the etiology of KD is still unclear. There are several hypotheses regarding the outbreak of KD, including infection, genetics, and immunity. One hypothesis is that KD is caused by an infectious agent that causes an autoimmune disease in only genetically predisposed individuals, and a series of immune responses are expressed in vasculitis (4–6). Immunohistochemical analysis shows accumulation in vasculitis lesions with many monocytes and macrophages and presence of CD8 T lymphocytes and IgA plasma cells (7, 8). Activated monocytes and macrophages secrete the mediators interleukin 1β and tumor necrosis factor, which induce vascular endothelial cell damage (9, 10).

Several studies have shown that breastfeeding plays a crucial role in the composition of the immune system. It helps improve immunity (11, 12), and studies on its association with various diseases, such as inflammatory bowel disease (13) and asthma (14), are being conducted, encouraging breastfeeding worldwide. Breast milk contains multiple cytokines and maternal leukocytes, which promote phagocytosis and differentiation into dendritic cells (15). Human milk oligosaccharides, which account for a large portion of breast milk, maintain the intestinal microbiome and support the epithelial barrier function, resulting in protective effects on the development of mucosal immunity (16). Moreover, microRNAs and mammary stem cells are known as factors of breastmilk that affect the infantile immune system (17). However, studies on the effect of breastfeeding, which is deeply related to immunity, on the occurrence of KD, a systemic immune disease, are scarce. Previous studies in Japan (18), Germany (19) and China (20) have announced the protective effects of breastfeeding on KD. Still, all of them have been targeted at a small number of infants.

KD mainly occurs in children aged <5 years, and the prevalence rate is high in Northeast Asia, including Korea. According to a report by the Health Insurance Review & Assessment Service (HIRA) in 2016, the incidence of KD in Korea gradually increased between 2007 and 2014 (21). This trend was continuously maintained but decreased after the COVID-19 pandemic (22). This is due to the temporary decrease in respiratory infections in Korea due to the implementation of non-pharmaceutical interventions, such as mandatory mask-wearing, school closure, and testing and isolation of symptomatic individuals (23). In addition, there is a possibility that the incidence rate of KD seems to have decreased due to delayed diagnosis and hospital closure due to quarantine measures caused by COVID-19. However, the exclusive breastfeeding rate (EBR) decreased in Korea in a similar period. The EBR peaked in 2009 and decreased slightly in 2012 compared with that in 2009 (24). This trend gradually accelerated; EBR at 6 months was 11.4% in 2012 and sharply decreased to only 2.3% in 2015 (25). After observing these changes in the figures, we believe that human milk might contribute to the prevention of KD.

In this study, the incidence of KD according to the breastfeeding type was investigated in infants in Korea using nationwide cohort data to determine whether breastfeeding can reduce the incidence of KD and its complications. This study aimed to confirm the preventive effects and excellence of breastfeeding.

## Methods

### Data source

This study was conducted with the provision of the National Health Insurance Service (NHIS) in South Korea (NHIS-2021-1-636). The NHIS's database contains de-identified information, such as the Tenth Revision of the International Classification of Disease (ICD-10), prescription codes, procedures, operations, mortality records, and history of hospitalization. We merged NHIS data and the National Children's Health Examination (NCHE) data for this study. The NCHE is conducted for all infants and children aged <6 years. The NCHE began in November 2007 for health insurance subscribers, and since January 2008, it has expanded to the entire nation, including recipients of medical benefits. NCHE is conducted according to the age group (4–6, 9–12, 18–24, 30–36, 42–48, 54–60, and 66–71 months) and includes interviews with the primary caregivers and essential physical examinations. There is a tendency to start weaning food around 4–6 months in Korea (24, 26), so infant screening data between 4 and 6 months, including a questionnaire on breastfeeding, were used in this study.

Breastfeeding status was collected using the primary caregiver-answered questionnaire at 4–6 months of age of the infant. The feeding types were divided as follows: (1) exclusive breastfeeding, (2) exclusive formula feeding, (3) partial breastfeeding (mixed feeding with breast milk and formula milk), and (4) special milk formula feeding. A special milk formula is used for infants who cannot withstand conventional formula due to underlying diseases, such as milk allergy, eating problems, and mineral imbalance. In this study, infants fed with special formula were excluded because the underlying disease may have affected the occurrence of KD.

This study was approved by the Institutional Review Board of Hanyang University Hospital on April 29, 2021 (HYUH 2021-04-076). The requirement for informed consent was waived by the board.

### Study design and variables

We performed a retrospective cohort study using NHIS data and the NCHE database from January 1, 2008, to December 31, 2014. If the breastfeeding questionnaires were missing, infants who were fed special milk, infants diagnosed with KD before the NCHE, and infants who died before 3 years of age were excluded. We investigated infants admitted under the diagnosis of KD (ICD-10, M30.3). We analyzed young age, judging that the effect of breastfeeding on KD will decrease with age, and investigated the age at which no significant effect appeared by expanding the observation range yearly.

Demographic characteristics, such as sex, body measurement (height, weight, head circumference in the first NCHE, diagnosis date), birth weight, premature babies, and neonatal intensive care unit (NICU) admission, were investigated.

### Statistical analysis

A normality test was performed using the Anderson–Darling test for continuous variables. All continuous variables in this study showed a non-normal distribution and are presented as medians and the 25–75th percentiles. Categorical variables are presented as numbers and percentages. We identified variables that statistically differed between the groups with and without KD within 12 months of age using a univariable analysis. The Wilcoxon rank-sum test was performed to compare the two groups of continuous variables. Fisher's exact test was performed to compare categorical variables between the two groups. Next, we analyzed the association between KD and type of feeding using logistic regression analyses, and the crude odds ratios (ORs) and 95% confidence intervals (CIs) were calculated. After adjusting for covariates, the multivariable analysis showed a significant difference in the univariable analysis. Adjusted ORs (aORs) and 95% CIs were calculated using multivariable logistic regression analysis.

We extended the observation period to 24 and 36 months to determine the duration of the effects of breastfeeding on the development of KD. Statistical analysis was performed for the follow-up periods of 24 and 36 months, respectively, in the same way as the univariable and multivariable analyses for the follow-up period of 12 months. Statistical significance was determined using two-sided tests, with significance indicated by a *P-*value <0.05. All analyses were performed using SAS version 9.4 (SAS Institute Inc., Cary, NC, USA) and R version 3.5.2 (R Foundation for Statistical Computing, Vienna, Austria).

## Results

**Figure 1:**
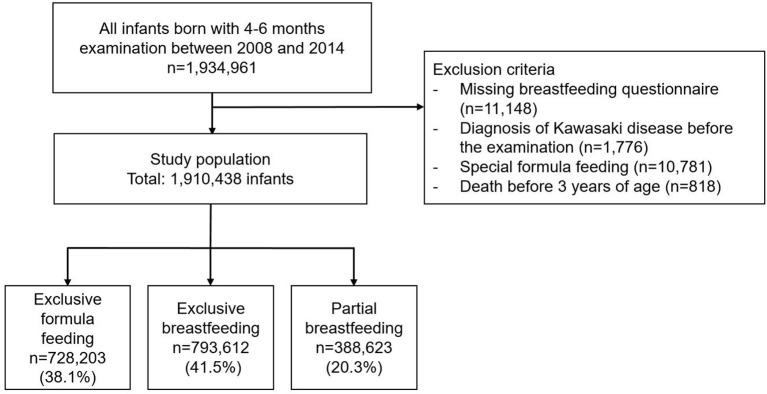
Flow diagram of cohort selection.

From 2008 to 2014, a total of 1,934,961 infants were examined for 4–6 months. The occurrence of KD was followed until 3 years of age in the examined children. Among them, 1,910,438 patients were finally included in this study, excluding those with missing breastfeeding questionnaire, infants who were diagnosed with KD before the examination, infants who were fed a special formula, and those who died before the age of 3 years. Moreover, 728,203 (38.1%) infants were exclusively formula fed and 793,612 (41.5%) infants were exclusively breastfed. A total of 388,623 (20.3%) infants were fed breast milk and formula milk (Figure 1).

More boys received exclusive formula feeding, while more girls received exclusive breastfeeding. Preterm infants and infants with more than 5 days of NICU admission were relatively more likely to receive formula feeding, but the birth weight did not differ among the three groups (Table 1).

**Table 1:** Demographic characteristics of the participants according to feeding type.

	**Total**	**Exclusive formula feeding (*n* = 728,203)**	**Exclusive breastfeeding (*n* = 793,612)**	**Partial breastfeeding (*n* = 388,623)**
Male (*n*, %)	985,904 (51.6)	386,117 (53.0)	389,439 (49.1)	210,348 (54.1)
Weight (kg), median [Q1–Q3]	8.0 [7.4–8.8]	8.1 [7.5–8.8]	8.0 [7.3–8.7]	8.0 [7.4–8.7]
Height (cm), median [Q1–Q3]	67.3 [65.3–69.2]	67.6 [65.6–69.6]	67.0 [65.1–69.0]	67.3 [65.3–69.3]
Head circumference (cm), median [Q1–Q3]	42.7 [41.7–43.7]	42.9 [41.9–44.0]	42.5 [41.5–43.5]	42.7 [41.7–43.7]
Prematurity (*n*, %)	67,493 (3.6)	37,113 (5.2)	16,516 (2.1)	13,864 (3.6)
NICU admission ≥ 5 days	110,845 (5.8)	52,151 (7.2)	36,305 (4.6)	22,389 (5.8)
Birth weight (kg), median [Q1–Q3]	3.2 [2.9–3.5]	3.2 [2.9–3.5]	3.2 [3.0–3.5]	3.2 [2.9–3.5]

NICU, neonatal intensive care unit.

At the age of 10–12 months, 3,854 infants (0.2% of the total) were diagnosed with KD, and the univariable analysis attributing factors are shown in Table 2. It can be seen that the ratio of infants with exclusive formula feeding is significantly higher in the KD group. There was a higher proportion of male patients with KD than those without KD, but the proportion of premature infants did not show a statistically significant difference. However, the prevalence of KD was high with NICU admission. There was no difference in the birth weight. Height, weight, and head circumference at the examination date tended to be higher at the time of examination.

**Table 2:** Univariable analysis of attributing factors for Kawasaki disease at 10–12 months of follow-up age.

	**No KD (*n* = 1,906,584)**	**KD (*n* = 3,854)**	*P*-value*
Type of feeding (*n*, %)			**<0.001**
Exclusive formula feeding	726,563 (38.1)	1,640 (42.6)	
Exclusive breastfeeding	792,150 (41.5)	1,462 (37.9)	
Partial breastfeeding	387,871 (20.3)	852 (19.5)	
Male (*n*, %)	983,497 (51.6)	2,407 (62.5)	**<0.001**
Weight (kg), median [Q1–Q3]	8.0 [7.4–8.8]	8.1 [7.5–8.8]	**0.001**
Height (cm), median [Q1–Q3]	67.3 [65.3–69.2]	67.6 [65.6–69.5]	**<0.001**
Head circumference (cm), median [Q1–Q3]	42.7 [41.7–43.7]	43.0 [42.0–44.0]	**<0.001**
Prematurity (*n*, %)	67,362 (3.6)	131 (3.4)	0.661
NICU admission ≥ 5 days	110,574 (5.8)	271 (7.0)	**0.001**
Birth weight (kg), median [Q1–Q3]	3.2 [2.9–3.5]	3.2 [2.9–3.5]	0.876

KD, Kawasaki disease; NICU, neonatal intensive care unit. *Anderson–Darling test for normality. Wilcoxon rank-sum test or Fisher's exact test for univariable analysis. Boldface indicates a statistically significant difference with P-value <0.05.

The risk of KD according to the feeding type for each observation period is shown in Table 3. Infants exclusively breastfed were less likely to develop KD until the age of 10–12 months. Even after adjusting for all covariates (sex, height, weight, head circumference, and NICU admission), the protective effect of breastfeeding against KD at the age of ≤ 1 year was clear; aORs for KD were 0.84 (95% CI, 0.78–0.90) for exclusive breastfeeding and 0.86 (95% CI, 0.79–0.94) for partial breastfeeding. At 22–24 months of age, only exclusive breastfeeding reduced the risk of KD; aORs for KD were 0.94 (95% CI, 0.90–0.98) for exclusive breastfeeding and 0.98 (95% CI, 0.92–1.03) for partial breastfeeding after adjusting for covariates (Supplementary Table 1). At 34–36 months of age, there was no difference in risk between groups; aORs for KD were 0.99 (95% CI, 0.95–1.02) for exclusive breastfeeding and 1.01 (95% CI, 0.96–1.05) for partial breastfeeding after adjusting for covariates (Supplementary Table 2).

**Table 3:** Analysis of the cumulative risk for breastfeeding and KD according to follow-up months.

	**Model 1: crude model**		**Model 2: multivariable model^a^**	
**Follow-up age**	**OR**	**95% CI**	**OR**	**95% CI**
**10–12 months**
Exclusive breastfeeding	**0.82**	**0.76–0.88**	**0.84**	**0.78–0.90**
Partial breastfeeding	**0.86**	**0.79–0.94**	**0.86**	**0.79–0.94**
**22–24 months**
Exclusive breastfeeding	**0.92**	**0.88–0.97**	**0.94**	**0.90–0.98**
Partial breastfeeding	0.98	0.93**–**1.03	0.98	0.92**–**1.03
**34–36 months**
Exclusive breastfeeding	0.97	0.94**–**1.01	0.99	0.95**–**1.02
Partial breastfeeding	1.01	0.97**–**1.06	1.01	0.96**–**1.05

OR, odds ratio; CI, confidence interval.

a Adjusted for sex, height, weight, head circumference, and neonatal intensive care unit admission at 10–12 months follow-up age. Adjusted for sex, height, head circumference, neonatal intensive care unit admission at 22–24 months follow-up age. Adjusted for sex, height, head circumference, and neonatal intensive care unit admission at 34–36 months of follow-up age. Boldface indicates a significant results with OR <1.

## Discussion

This is the first study to prove that breastfeeding has a protective effect against the occurrence of KD, using a large amount of national data. It was clearly shown that breastfeeding during the first 4–6 months of age could significantly reduce the incidence of KD at the age of <1 year, and it was confirmed that the protective effect of breastfeeding against KD remains up to 2 years of age.

In a case-control study previously conducted in Germany (19) and China (20), only 308 and 389 patients with KD, respectively, were studied. Since the study conducted in Germany aimed to prevent KD when using vitamin D and breastfeeding together, it cannot be said that it objectively showed the excellence of breastfeeding, as it does not include all cases with more than 2 weeks of breastfeeding. A case-control study in China showed that the protective effect of exclusive breastfeeding against KD was excellent with univariable OR of 0.55 and multivariable OR of 0.53. Because this was a single-center study, a more detailed analysis was possible. Subgroup analysis was performed for incomplete KD, IVIG responsiveness, and presence of coronary aneurysms, and all subgroups showed significant results with OR <1. However, in the case of partial breastfeeding, compared to formula feeding, there was a possibility of biased intervention, and the memory was possibly distorted because it confirmed whether breastfeeding was performed through a phone call with a guardian after more than 3 years. A nationwide survey in Japan (18), which investigated the relationship between breastfeeding and KD, also showed the effectiveness of breastfeeding (multivariable OR, 0.45 for exclusive breastfeeding at 6–7 months of age). However, this study included only 232 actual patients with KD, and only 3.3% of all infants received formula feeding. As the number of groups that did not breastfeed was extremely small, the effect of breastfeeding was somewhat emphasized. Recently, in a study introducing the advantages of breastfeeding in Korea (27), the number of patients rapidly decreased through the propensity score matching process, and accordingly, the protective effect of breastfeeding was not shown.

Compared with these studies, our study contains far more research subjects. Nevertheless, the ratio of breastfeeding to formula feeding is proportional, and since the examination was conducted at the time of breastfeeding, a relatively accurate form of breastfeeding can be identified. Moreover, the results can be trusted because the results of partial breastfeeding are in the middle of the results of exclusive breastfeeding and exclusive formula feeding, and the preventive effect of breastfeeding is maintained until 2 years of age and decreases with age.

The epidemiological characteristics of KD strongly support ubiquitous agents, which generally result in asymptomatic infection but cause KD in a small subset of genetically predisposed children (6). Moreover, the prevalence of CD8 T cells in the inflammatory infiltrate and the upregulation of cytotoxic T cell and interferon pathway genes in the coronary arteries of children who have died of KD highly indicate a viral etiology (28). Transforming growth factor (TGF)-β, which affects T cell regulation, plays a role in inducing neoangiogenesis, cardiomyocyte hypertrophy, calcification, and fibrosis in the cardiovascular system (29). TGF-β may play an important role in KD pathogenesis, as genetic variations in TGF-β can affect KD sensitivity, disease outcomes, and response to treatment (30). TGF-β in human milk promotes IgA production, aids mucosal recovery of the neonatal gastrointestinal tract, acts as an additional factor producing immunomodulatory immune responses, and affects the neonatal gut microbiome (31). Breast milk contains immunological memory substances, such as secretory immunoglobulin A, lactoferrin, nucleotides, and oligosaccharides, and various nutrients, hormones, and growth factors (including TGF-β) that help in the development of the immune system (32). In addition, diverse factors, such as allergens, regulate and promote immune responses, and several factors help balance the intestinal microbiota (33). In conclusion, breastfeeding increases resistance to infection and promotes an anti-inflammatory response compared to formula feeding. Accordingly, the American Academy of Pediatrics strongly recommends breastfeeding for 6 months before starting complementary foods and recommends continuing breastfeeding for as long as possible (34).

The mechanism by which breastfeeding plays a protective role in the outbreak of KD is still unclear; however, some hypotheses based on the effects of breastfeeding are as follows: First, the immune enhancement effect of breast milk reduces the incidence of autoimmune diseases and suppresses abnormal immune responses, such as KD. It is hypothesized that various immunological memory substances in breast milk prevent abnormal immune responses (35). In addition, breast milk can help the immune system mature and minimize damage from abnormal immune responses (32). Second, breastfeeding matures the immune system and suppresses the known risk factors for KD, such as infection. Several studies have shown that breast milk has a protective effect against infectious diseases. Since the representative trigger point of KD is infection, it is hypothesized that suppressing the expression of infectious diseases through breastfeeding can suppress the onset of KD.

As this study was conducted with national data, several restrictions exist. First, the date of the first NCHE is wide, between 4 and 6 months of age, so the exact age is unknown, and the breastfeeding period is unknown. Second, because it was a study based on claims data, clinical information, such as fever period, echocardiographic findings, and presence or absence of coronary aneurysm, could not be obtained. Moreover, since we selected patients using the ICD diagnostic criteria, the diagnosis may not be accurate because of the nature of KD diagnosis based on the clinical criteria.

In conclusion, using extensive national data, we found that breastfeeding can reduce the incidence of KD. This study has a distinct advantage because it was based on many subjects compared to previously published studies. In addition, the effect of breastfeeding on KD disappeared before the age of 2 years, but considering that the age of the outbreak was around one of the ages, it is thought that it could serve as the basis for active recommendation of breastfeeding.

## Data availability statement

The original contributions presented in the study are included in the article/Supplementary material, further inquiries can be directed to the corresponding author.

## Ethics statement

The studies involving human participants were reviewed and approved by Hanyang University Hospital. Written informed consent from the participants' legal guardian/next of kin was not required to participate in this study in accordance with the national legislation and the institutional requirements.

## Author contributions

YK had full access to all the data in the study and takes responsibility for the integrity of the data and the accuracy of the data analysis. Conceptualization and formal analysis: JN, YC, and YK. Investigation and statistical analysis: JN, YC, and JL. Writing–original draft preparation: JN, YC, JL, and YK. Writing–review and editing and funding acquisition: JN, SY, and YK. All authors contributed to the article and approved the submitted version.

## Funding

This work was supported by the Hanyang University Pediatrics Alumni Scholarship. The funding organization had no role in the design and conduct of the study, collection, management, analysis, and interpretation of the data, preparation, review, or approval of the manuscript, or decision to submit the manuscript for publication.

## Conflict of interest

The authors declare that the research was conducted in the absence of any commercial or financial relationships that could be construed as a potential conflict of interest.

## Publisher's note

All claims expressed in this article are solely those of the authors and do not necessarily represent those of their affiliated organizations, or those of the publisher, the editors and the reviewers. Any product that may be evaluated in this article, or claim that may be made by its manufacturer, is not guaranteed or endorsed by the publisher.
