# Supplementary material for: Immune-modulatory effect of human milk in reducing the risk of Kawasaki disease: A nationwide study in Korea
Source: Front Pediatr. 2022 Sep 8;10:1001272. doi: 10.3389/fped.2022.1001272 (PMC9492926; doi:10.3389/fped.2022.1001272)
Supplement: Supplementary file 1 [file Data_Sheet_1.docx]

Table 1; online. Univariable analysis of attributing factors for Kawasaki disease at 22–24 months of follow up age

|  | No KD  (n=1,900,324) | KD  (n=10,114) | P-value |
| --- | --- | --- | --- |
| Type of feeding (n, %)  Exclusive formula feeding  Exclusive breastfeeding  Partial breastfeeding | 724,202 (38.1)  789,583 (41.5)  386,539 (20.3) | 4,001 (38.5)  4,029 (39.8)  2,084 (20.6) | **0.001*** |
| Male (n, %) | 979,849 (51.6) | 6,055 (59.9) | **<0.001*** |
| Weight (kg), median [Q1–Q3] | 8.0 [7.4–8.8] | 8.0 [7.4–8.8] | 0.205 |
| Height (cm), median [Q1–Q3] | 67.3 [65.3–69.2] | 67.5 [65.5–69.4] | **<0.001*** |
| Head circumference (cm), median [Q1–Q3] | 42.7 [41.7–43.7] | 43.0 [42.0–44.0] | **<0.001*** |
| Prematurity (n, %) | 67,114 (3.6) | 379 (3.8) | 0.274 |
| NICU admission ≥ 5 days | 110,154 (5.8) | 691 (6.8) | **<0.001*** |
| Birth weight (kg), median [Q1–Q3] | 3.2 [2.9–3.5] | 3.2 [2.9–3.5] | 0.681 |

Abbreviations: NICU, neonatal intensive care unit

*Anderson-Darling test for normality. Wilcoxon rank-sum test or Fisher’s exact test for univariable analysis

Boldface indicates a statistically significant difference with *P*-value *<0.05.*

Table 2; online. Univariable analysis of attributing factors for Kawasaki disease at 34–36 months of follow-up age

|  | No KD  (n=1,894,520) | KD  (n=15,918) | P-value |
| --- | --- | --- | --- |
| Type of feeding (n, %)  Exclusive formula feeding  Exclusive breastfeeding  Partial breastfeeding | 722,077 (38.1)  787,116 (41.5)  385,327 (20.3) | 6,126 (38.5)  6,496 (40.8)  3,296 (20.7) | 0.159 |
| Male (n, %) | 976,526 (51.6) | 9378 (58.9) | **<0.001*** |
| Weight (kg), median [Q1–Q3] | 8.0 [7.4-8.8] | 8.0 [7.4-8.8] | 0.144 |
| Height (cm), median [Q1–Q3] | 67.3 [65.3-69.2] | 67.4 [65.5-69.3] | **<0.001*** |
| Head circumference (cm), median [Q1–Q3] | 42.7 [41.7-43.7] | 42.9 [42.0-43.9] | **<0.001*** |
| Prematurity (n, %) | 66,928 (3.6) | 565 (3.6) | 0.973 |
| NICU admission ≥ 5 days | 109,806 (5.8) | 1039 (6.5) | **<0.001*** |
| Birth weight (kg), median [Q1–Q3] | 3.2 [2.9-3.5] | 3.2 [2.9-3.5] | 0.505 |

Abbreviations: NICU, neonatal intensive care unit

*Anderson-Darling test for normality. Wilcoxon rank-sum test or Fisher’s exact test for univariable analysis

Boldface indicates a statistically significant difference with *P*-value *<0.05.*
